# Prion Replication in the Hematopoietic Compartment Is Not Required for Neuroinvasion in Scrapie Mouse Model

**DOI:** 10.1371/journal.pone.0013166

**Published:** 2010-10-05

**Authors:** Corinne Loeuillet, Catherine Lemaire-Vieille, Philippe Naquet, Marie-France Cesbron-Delauw, Jean Gagnon, Jean-Yves Cesbron

**Affiliations:** 1 Laboratoire Adaptation et Pathogénie des Micro-organismes, Centre National Recherche Scientifique UMR 5163, Université Joseph Fourier, Grenoble, France; 2 Centre d'Immunologie de Marseille-Luminy, Institut National de la Santé et de la Recherche Médicale, Centre National Recherche Scientifique, Université de La Méditerranée, Marseille, France; Universidad de la Republica, Uruguay

## Abstract

Fatal neurodegenerative prion diseases are caused by the transmissible PrP^Sc^ prion agent whose initial replication after peripheral inoculation takes place in follicular dendritic cells present in germinal centers of lymphoid organs. However, prion replication also occurs in lymphoid cells. To assess the role of the hematopoietic compartment in neuroinvasion and prion replication, we generated chimeric mice, on a uniform congenic C57/BL6J background, by bone marrow replacement with hematopoietic cells expressing different levels of PrP protein. Nine different types of chimeric mice were inoculated intraperitoneally either with the lymphotropic Rocky Mountain Laboratory (RML) strain or the non lymphotropic ME-7 scrapie strain, at different doses. Here, we clearly demonstrate that overexpression of PrP by the hematopoietic system, or the lack of PrP expression by the bone marrow derived cells, does not change the incubation time period of the disease, even when the mice are infected at limiting doses. We conclude that the hematopoietic compartment is more or less permissive to prion replication, both for RML and ME-7, but does not play a role in neuroinvasion.

## Introduction

After oral exposure to prions, accumulation of infectivity is first detected in mucosal lymphoid organs. Neuroinvasion occurs later, and involves the translocation of PrP^Sc^ *via* peripheral nerves and its accumulation in the brain. PrP deficient mice are not susceptible to prion [1] and the expression levels of PrP^c^ protein correlate inversely with prion disease incubation time and disease progression [2].

The role of the immune system in prion diseases has been suggested when it was observed that severe combined immunodeficient mice, which lack B and T lymphocytes, are resistant to peripheral prion inoculation, but susceptibility can be restored after bone marrow (BM) transplantation [3], [4]. From these original observations, several studies have been carried out to characterize the cell types involved in agent replication before neuroinvasion.

There is a general agreement that follicular dendritic cells (FDCs) are the principal sites for amplification of PrP^Sc^ in lymphoid tissues during the early phase of infection, before the disease spreads to the nervous system [5], [6], [7]. FDCs are present in follicles of any secondary lymphoid organ and belong to the stromal cells compartments. Recent data on mesenchymal precursor cells from the peripheral blood, suggest a close relationship between FDCs and fibroblast-like cells [8]. The immune system allows the differentiation and maintenance of FDC network in lymphoid organs by the secretion of cytokines such as TNFα and lymphotoxins α and β by B cells.

ME-7 and RML strains are the two principal mouse inocula, which have been used in mouse scrapie models. Although RML and ME-7 neuroinvasion is dependent upon the presence of FDCs, these two strains present differences in affinity for bone marrow (BM) derived cells. Following infection with RML strain, high levels of infectivity accumulate in spleen in the absence of PrP^c^ expression by FDCs so long as PrP^c^ is expressed by hematopoietic derived cells, suggesting the lymphotropic nature of the RML strain [9], [10]. Exactly opposite result has been reported using ME-7 strain as in this case, no infectivity accumulate in spleen in the absence of PrP^c^ expression by FDCs even if the hematopoietic cells express PrP^c^ [11],[12].

The question is whether prion replication by BM derived cells is involved in neuroinvasion. For that, we have carried out experiments using mice on a uniform congenic C57/BL6J background, reconstituted after lethal irradiation with BM from three groups of mice expressing different level of PrP^c^: (i) mice where *prp* gene has been deleted (Prp^0/0^ [1]), (ii) wild type mice, and (iii) mice carrying several copies of *prp* gene (Tga20 mice [2]). These mice express 0, 1 or 4–5 times the level of PrP^c^ respectively, relative to wild type mice. The animals were inoculated either with lymphotropic RML strain or ME-7 scrapie strain.

In this work, we clearly show that the level of PrP^c^ expression in the hematopoietic compartment does not influence the time course of the induced disease. Indeed the mice reconstituted with BM from Prp^0/0^ mice have the same incubation time as mice reconstituted with BM from wild type mice, or from mice overexpressing PrP^c^, even when inoculated with limiting prion doses. Although ME-7 strain has been described as a non lymphotropic strain, we observed infectivity in the spleen of PrP^0/0^ mice reconstituted with BM overexpressing PrP^c^. These results indicate the fact that a cell derived from hematopoietic compartment can replicate both ME-7 and RML scrapie strain, but cannot account for neuroinvasion.

## Results

### PrP^c^ expression by BM derived cell does not influence the scrapie incubation period in chimeric mice

Each set of Prp^0/0^, Tga20 or B6 congenic mice were reconstituted with femoral BM from the three same sources. These combinations led to the generation of nine different types of chimeric mice for a total of more than 120 animals. We use the following convention to name those mice groups: when we write Tga20→B6 mice, this means that Tga20 BM cells have been injected in B6 mice for hematopoietic reconstitution.

In a first set of experiment, we injected intraperitoneally 100 µl of a ME-7 inoculum containing 10^−5^ LogLD50. Wild-type B6 inoculated mice died after 252 days (IQR, 237–267 days) post-IP inoculation. As expected, the inoculated Tga20 mice have a shorter incubation period 130 days (IQR, 123–135 days) than the B6. This is explained by the fact that PrP^c^ expression is 5 to 6 times higher in Tga20 mice than B6 wild type animals [2]. The chimeric mice on the B6 background (B6→B6, n = 4; Tga20→B6, n = 6 and the Prp^0/0^→B6, n = 2) presented the same incubation period (261 days; IQR, 232–295 days/278 days; IQR, 234–315 days/260 days, respectively) as wild-type B6. Similarly the incubation period of the chimeric mice on the Tga20 background were not significantly different than the Tga20 mice, whatever the origin of the BM used for the reconstitution: Tga20→Tga20, n = 6, 140 days (IQR, 124–158 days), B6→Tga20, n = 7, 144 days (IQR, 116–161 days), and Prp^0/0^ →Tga20, n = 4, 150 days (IQR, 139–155 days).

**Figure 1:**
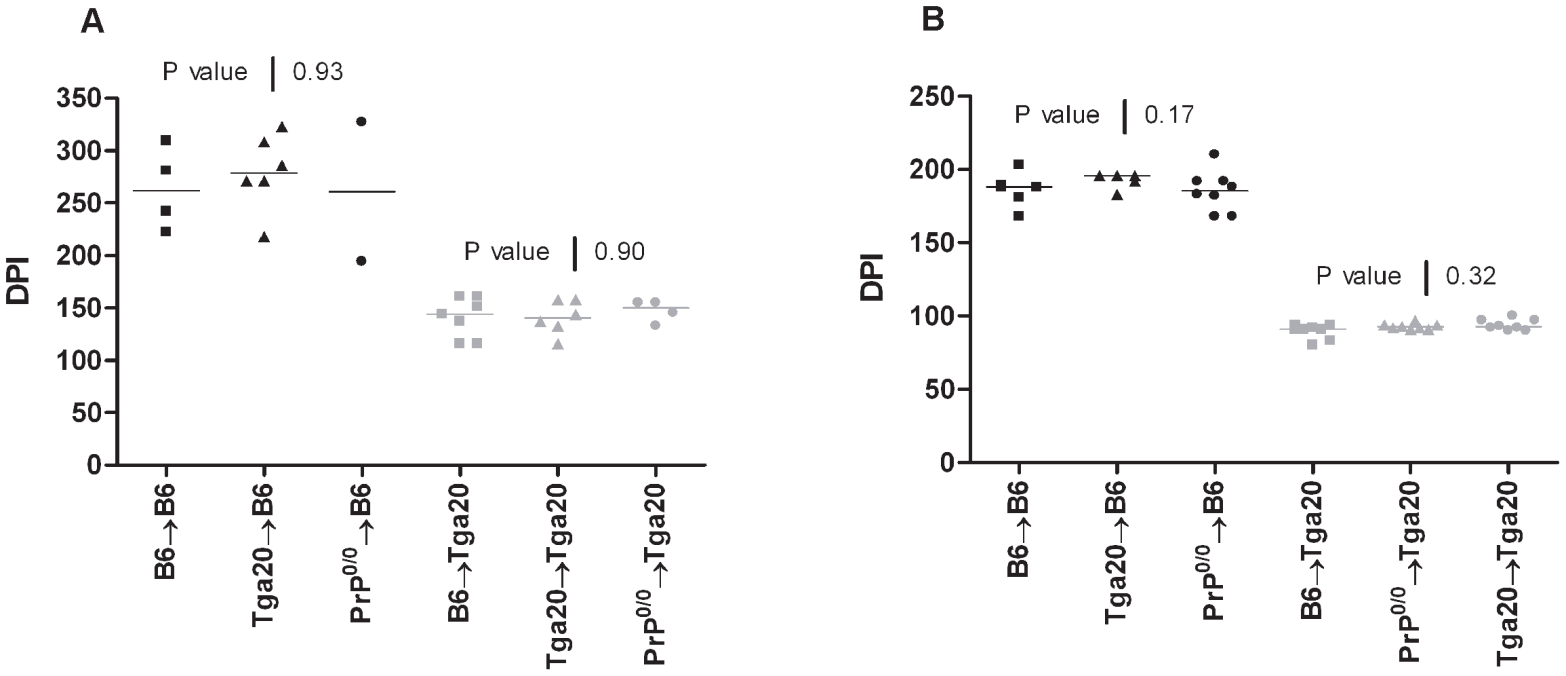
PrP^c^ overexpression by the hematopoietic system does not play a role in neuroinvasion. B6 (black) or Tga20 (grey) mice were lethally irradiated, reconstituted with femoral bone marrow cells from B6 (square), Tga20 (triangle) or Prp^0/0^ (circle) mice and inoculated either with the ME-7 (10^−5^ LogLD50) (A) or RML (10^−4^ LogLD50) (B) prion scrapie strains. The incubation periods are expressed as days post-inoculation (DPI). *P* values were obtained using the Kruskal-Wallis ANOVA test. In panel (A), *P* value of 0.93 was determined for the reconstituted B6 mice group and of 0.9 for the reconstituted Tga20 group. In panel (B), the *P* values were of 0.17 and of 0.32 for reconstituted B6 and Tga20 mice groups, respectively.

When injected with the lymphotropic RML strain (10^−4^ LogLD50) (Fig. 1b), wild-type B6 inoculated mice died after 201 days (IQR, 192–207 days) post-IP inoculation. Similar incubation times were observed in the chimeric mice B6→B6, n = 5, 188 days (IQR, 174–196 days), in the Tga20→B6 mice, n = 5, 196 days (IQR, 183–196 days), and in the Prp^0/0^→B6, n = 8, 185 days (IQR, 175–192 days). The Tga20 mice died after 99 days (IQR, 94–106 days) post-IP inoculation, compared to 92 days (IQR, 91–97 days) for the chimeric Tga20→Tga20 (n = 8), 91 days (IQR, 87–93 days) for the B6→Tga20 mice (n =  8) and 92 days (IQR, 91–94 days) for Prp^0/0^→Tga20 (n = 8). As expected, none of the chimeric mice harboring a Prp^0/0^ genetic background developed a clinical disease, for lack of expression of PrP^c^ protein [1].

From these experiments, we could conclude that the expression level of the PrP^c^ by the hematopoietic cells does not influence the scrapie incubation period. However it has been suggested that, when using high doses of inoculum, the PrP^Sc^ might be able to bypass the lymphoreticular system and invade directly the peripheral nervous system [3], therefore the PrP^c^ expression of BM derived cells would have little influence on the incubation time. In agreement with this idea, amplification of infectivity in PrP positive BM derived cells might be necessary in order to achieve neuroinvasion after inoculation with lower doses of prions.

**Figure 2:**
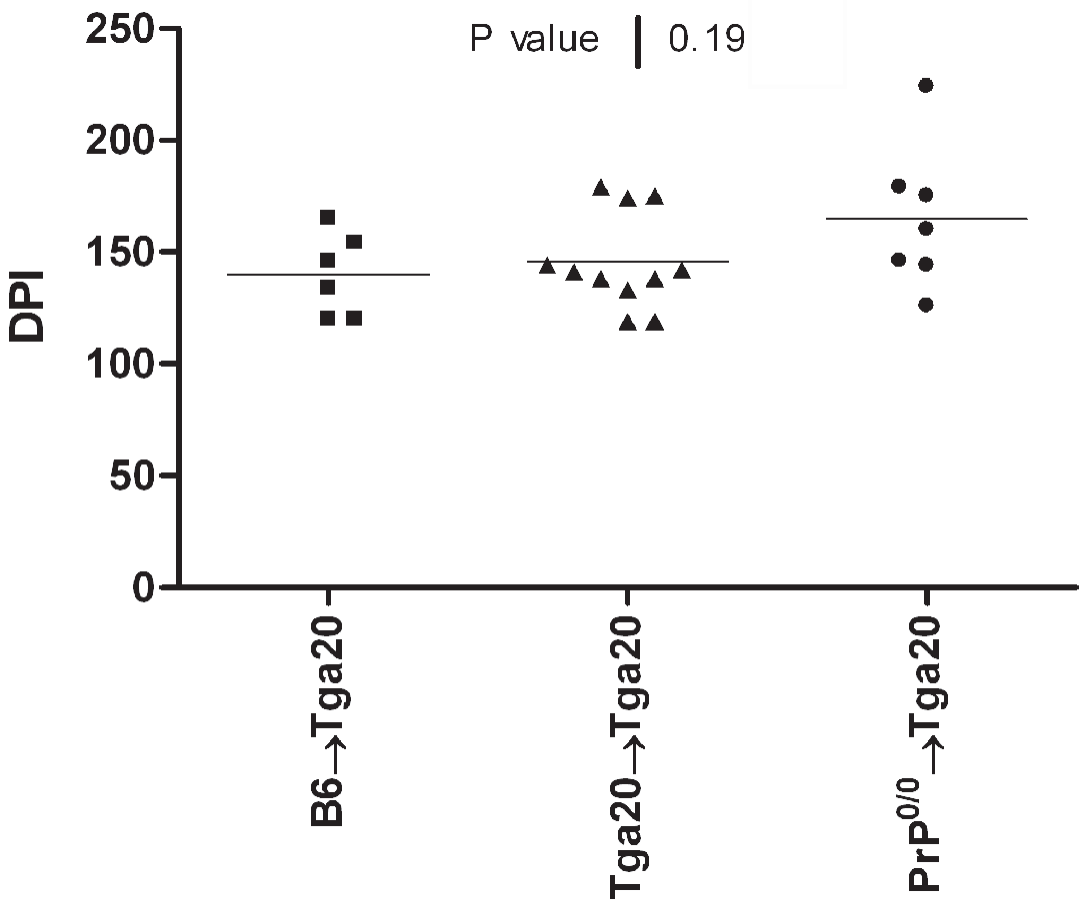
No difference in disease incubation period is observed in Tga20 chimeric mice inoculated with limiting doses of the RML strain (10^−7^ LogLD50). Tga20 mice were lethally irradiated, reconstituted with femoral bone marrow cells from B6 (square), Tga20 (triangle) or Prp^0/0^ (circle) mice and inoculated with the RML prion scrapie strains. The incubation periods are expressed as days post-inoculation (DPI). At this low dose, only 7/10 mice developed scrapie. A *P* value of 0.19 was obtained using the Kruskal-Wallis ANOVA test when comparing the three groups of mice.

To test this hypothesis we have inoculated intraperitoneally the chimeric mice on the Tga20 background with limiting doses of the lymphotropic RML strain (10^−7^ LogLD50). Accumulation of the RML strain in lymphoid organs has been extensively described [9], [10], in contrast to the ME-7 strain [11], [12]. If RML amplification by BM derived cells is required for neuroinvasion, therefore we would expect to observe a difference in the incubation period between Tga20 mice reconstituted with BM overexpressing PrP^c^ and Tga20 mice reconstituted with BM sampled from Prp^0/0^ mice. As shown in Fig. 2, no difference in incubation period was observed whatever the origin of the BM used for reconstitution: B6→Tga20, n = 6, 140 days (IQR, 120–165 days), Tga20→Tga20, n = 11, 139 days (IQR, 119–179 days), and Prp^0/0^→Tga20, n = 7, 160 days (IQR, 126–224 days). Since the amount of inoculum was very low, three inoculated mice did not develop clinical disease, while the seven mice that developed scrapie showed longer and more dispersed incubation periods than mice inoculated with high infective doses (Fig. 1). These results demonstrate that the replication of lymphotropic RML strain in BM derived cells does not play a critical role in neuroinvasion.

### ME-7 infectivity can be detected in the spleen of PrP^0/0^ mice reconstituted with Tga20 BM

Since RML strain replicates in BM derived cells [13] while ME-7 does not [5], the spleens of PrP^0/0^ chimeric mice were analyzed for the presence of prion infectivity. As expected, PrP^0/0^ chimeric mice did not develop a clinical disease, and therefore have been sacrificed at the end of the experiments (450 days post-inoculation). Because low levels of infectivity could not be detected by western blotting analysis, we performed a bioassay by intracerebral inoculation of Tga20 mice with these spleen extracts.

**Figure 3:**
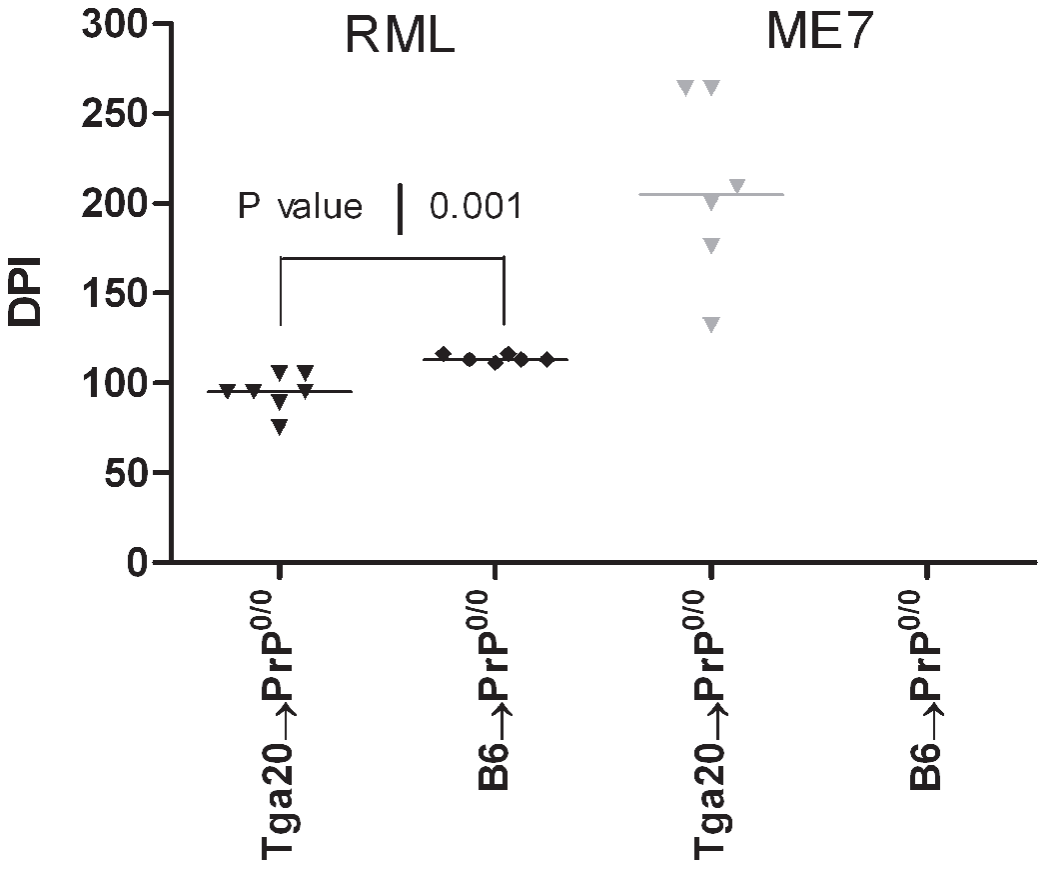
PrP^c^ overexpression by the hematopoietic cells favors prion agent replication in the spleen of reconstituted Prp^0/0^ mice. Spleen of Prp^0/0^ mice lethally irradiated reconstituted with femoral bone marrow cells from Tga20 (inverse triangle) or B6 (circle) mice and inoculated with RML (black) or ME-7 (grey) strains were sampled 450 days post-inoculation and subsequently inoculated to Tga20 mice. Days post-inoculation (DPI) are represented. A *P* value of 0.001 was obtained using the Mann-Whitney t-test when comparing the Tga20→Prp^0/0^ and B6→Prp^0/0^ mice.

Tga20 mice inoculated with spleen extract from both Tga20→Prp^0/0^ (n = 7) and B6→Prp^0/0^ (n = 6) died of scrapie after 95 days (IQR, 75–105 days) and 113 days (IQR, 111–116 days) post-inoculation, respectively (Fig. 3). These results corroborate reported data [13]. In contrast, no Tga20 mice infected with a crude extract from the spleen of B6→Prp^0/0^ ME-7 infected mice developed the disease after 450 days post-inoculation (n = 6). This is in agreement with previous data [5]. However, 6/6 Tga20 mice inoculated with the spleen of Tga20→Prp^0/0^ infected mice have developed a typical clinical disease, with accumulation of PrP^Sc^ in brain (data not shown). This indicates that ME-7 strain can replicate in the hematopoietic compartment at a low level, but only when BM derived cells overexpress PrP^c^.

## Discussion

Prion diseases are caused by a conformational change in widely expressed PrP^c^ protein, leading to the formation and accumulation of PrP aggregates. Although prion diseases cause degeneration of the central nervous system, the presence of infectivity can be detected in lymphoid tissues at a very early stage of the disease after peripheral inoculation [14]. Studies have shown that severely immunodeficient mice lacking B and T lymphocytes are resistant to peripheral prion infection, but susceptibility can be restored following BM transplantation [3], [4]. Further studies point out the prominent role of FDCs, which are not BM derived cells, in the initial replication of prion [5], [6], [7]. Even if infectivity has been demonstrated in lymphoid organs and blood, the role of the hematopoietic compartment still remains unclear. The question is whether prion replication by BM derived cells is involved in neuroinvasion.

Besides differences in scrapie strains used, interpretation of these sophisticated experiments is complicated by variations in the amounts of inoculum, and the genetic background of the mice utilized [15]. The sanitary status might also interfere with interpretation of the results. As a matter of fact, mice infection with specific pathogen and/or opportunistic agents could lead to chronic inflammation that is known to modify prion infection [16]. Similarly the fact that in all the studies published the mice were not on a congenic background could not guarantee a full histocompatibility situation.

In order to avoid possible graft host reaction, our strategy was to create chimeric mice on the B6 background expressing different levels of PrP^c^ in the hematopoietic compartment. In addition we performed embryo transfers to obtain animals devoid of pathogens, to limit inflammatory chronic infection that could interfere with the pathophysiology of the disease [16]. After lethal irradiation, Prp^0/0^, Tga20 or C57/BL6J congenic mice were reconstituted with BM from each of the other mice to yield nine different types of chimeric animals. The chimeric mice models used in this study were not designed to assess the role of FDCs. These cells do not derive from hematopoietic precursors, and it has been clearly observed that mice defective for FDCs present a delay in the development of the clinical diseases, showing that the initial replication of infectivity in FDCs is critical for neuroinvasion [5], [6], [7]. Nevertheless some Prp^Sc^ strains such as RML show lymphotropism and their infectivity may involve a contribution of hematopoietic cells [9], [10]. In this situation the level of PrP expression by hematopoietic cells should influence the incubation period. Conversely, no effect should be expected when using ME-7, a non lymphotropic strain [11],[12].

PrP^c^ is widely expressed in various types of tissues and cells, including hematopoietic stem cell [17]. Tga20 mice that carry 60 copies a ‘half genomic’ sequence of the prion protein gene, express approximately 5–6 fold higher levels of PrP^c^ in the central nervous system. In these mice, the PrP^c^ overexpression has also been observed both in CD3 positive thymocytes [18] and splenocytes [18], [19], [20] indicating that Tga20 BM derived cells overexpressed PrP^c^.

**Figure 4:**
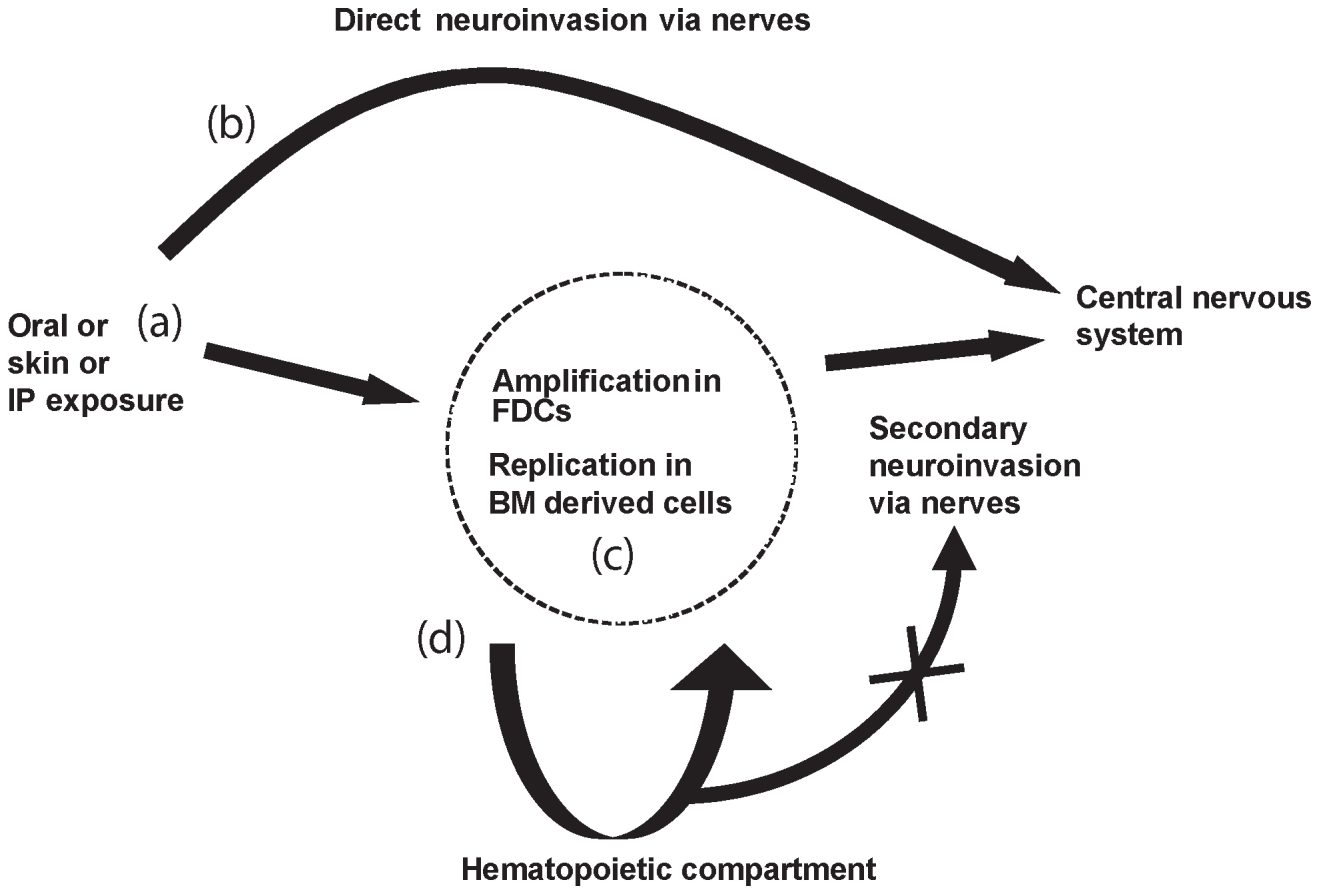
Routes of prion neuroinvasion after peripheral exposure. (a) Natural prion diseases are often acquired *via* peripheral exposure such as orally, or through skin lesions. How prion reaches its peripheral targets is not known. (b) Direct invasion of the central nervous system might occur with high doses of prion or exposure to neuroinvasive strains. (c) Whereas after exposure to limiting doses of infectivity or less neuroinvasive strains, replication in FDCs in the germinal centers of local lymphoid tissues might be necessary prior to neuroinvasion via closely associated nerve fibers. FDCs are dependent on the presence of B lymphocytes for maturation signals, such as lymphotoxin. (d) Haematogenous spread of infectivity *via* circulating bone marrow derived cells would not play a role in direct neuroinvasion.

Using high doses of either ME-7 or RML scrapie agents, we observed that the PrP status of the hematopoietic compartment did not modify the incubation time of the disease. These results are congruent with some previous partial data [21], [22]. To explain this, it has been proposed that high doses of inoculums may bypass the lymphoreticular system and directly invade the central nervous system via peripheral nerves, with no amplification in lymphoid tissues [3]. However, when chimeric mice were inoculated with limiting doses of RML, we observed that the level of PrP^c^ expression by BM derived cells did not affect the time course of scrapie infection. Therefore it is clear that the replication of prion in the hematopoietic compartment has no influence on neuroinvasion, even when the strain accumulates in lymphoid cells. This could indicate, as previously reported [23], that circulating cells are unlikely to play a role in neuroinvasion. However this does not exclude that cells such as dendritic cells could spread infectivity to other cells or to peripheral nerves, which in turn are involved in neuroinvasion [24]. If BM cells do not play a significant direct role in neuroinvasion, in situations such as contaminated blood products, these cells could transfer infectivity as previously reported [25]–[26]. As a summary, possible routes of prion neuroinvasion after peripheral exposure have been schematized in Fig. 4.

An unexpected result from this study concerns the paradigm of Brown and Blutter. When inoculated with lymphotropic RML strain, wild-type bone marrow cells transplanted in PrP deficient mice can restore accumulation and replication of prion in spleen, indicating that cells other than FDCs can replicate prion in the secondary lymphoid tissues [9]. By contrast Brown *et al.* reported a diametrically opposite outcome of similar experiment when reconstituted mice were inoculated with ME-7 strain [5]. We confirm these data; however we have evidenced infectivity in the spleen tissue of chimeric mice reconstituted with BM derived cells sampled from Tga20, when the ME-7 infectivity titer was low. This demonstrates that both ME-7 and RML can replicate in cells derived from hematopoietic compartment, other than FDCs.

## Methods

### Ethics Statement

Animals were housed according with the French Ethical Committee (Decree 87–848) and European Community Directive 86/609/EEC. Experiments were carried out under the supervision of JYC (agreement n° 38 05 17) in the animal care facilities approved by the Direction des Services Vétérinaires de l'Isère (N° A 38 516 10006). Before surgical procedure and prion inoculation, mice were anesthetized with a mixture injected intra-peritoneally of ketamine hydrochloride (Imalgen 500, Merial, 25 mg/kg body weight) and xylasine (Rompun, Bayer Healthcare, 12.5 mg/kg body weight).

### Mice

In order to circumvent tolerance problems after hematopoietic transfer, the speed congenic technology was used to generate mice with the same C57/BL6J genetic background for the two following strains: Prp^0/0^ prion deficient mice [1], and Tga20 mice [2] that were obtained from Pr. Charles Weissmann (Scripps Institute, Florida). The technology consists in using genetic markers throughout the genome to speed up ‘recovery’ of the recipient genome in the backcrossing phase of the construction of a congenic strain as described [27]. Tga20 mice, Prp^0/0^ and C57/BL6J, hereafter called B6 (Charles River Laboratories, Lyon), were housed in ventilated cages and maintained under specific pathogen-free conditions. Scrapie inoculated mice were housed in a biosafety laboratory level 3 animal care facility in cages placed in ventilated and negative pressure insulator.

### Bone marrow chimeras

Chimeric mice (>120) were reconstituted by injecting into the tail vein 5 to 10×10^6^ femoral BM cells into lethally irradiated (9.5 Gy) recipient 4 weeks old mice. B6 mice were reconstituted with BM cells from either B6, Tga20 or Prp^0/0^ mice; Tga20 mice reconstitution was performed with Tga20, B6 or Prp^0/0^ BM cells; and Prp^0/0^ were reconstituted with BM sampled from Tga20, B6 or Prp^0/0^ mice. The day following reconstitution, mice were treated with ciprofloxacine (0.1 mg/ml in drinking water) for ten days. Successful hematopoietic reconstitution was assessed 2 months after engraftment: CD3, CD4, CD8 lymphocytes total numbers and PrP^c^ expression by these cells were determined by flow cytometry analysis (data not shown). Failure led to the death of the mice within two weeks following irradiation.

### Source of the scrapie agent and inoculation

The ME-7 and the RML prion strains were maintained by successive inoculations into B6 mice. The scrapie inocula were prepared from brain tissues collected from terminally sick mice. The brain homogenate was prepared in PBS (10% w/v), and the presence of PrP^Sc^ confirmed by western blot analysis as described in [28]. More than 100 mice were successfully reconstituted and intraperitoneally inoculated with 100 µl of inoculum. Endpoint titrations were performed as described in [10].

### Measurement of the incubation period

For ethical reasons, the mice were sacrificed at the onset of the disease rather then waiting for their death. The onset was defined by the clear appearance of at least three of the following neurological symptoms: trembling, prostration, feet clasping when lifted, increased tone of the tail. The incubation period was taken as the time from inoculation to the euthanasia of the mice. Mice were monitored three times a week, beginning two months after inoculation. Tissues were collected, and frozen (−80°C) for subsequent western blot analysis, or reinoculation into Tga20 mice. Incubation period data was expressed as median and Inter Quartile Range (IQR). Differences in incubation periods were tested by the Kruskal-Wallis ANOVA test (Fig. 1 & 2) or the Mann-Whitney t-test (Fig. 3). In all comparisons, the level of significance was set at 0.05.
